# Abstracts and Selections

**Published:** 1915-08

**Authors:** 


### Abstracts and Selections.

### The Need for Greater Care in Surgical Diagnosis.*

BY DR. A. E. SWEATLAND, NACOGDOCHES, TEXAS.

The importance of the internist is emphasized and no surgeon should fail to avail himself of the same at all times. The lack of due diligence in making diagnosis in surgical cases is brought out, suggesting that many times operation would be decided against if further consideration were given the case in hand. While not discarding the exploratory operation entirely, with more diligent care in diagnosis, it would be reduced to the minimum. Attention is called to the responsibility of the surgeon in making a final diagnosis, and he really becomes a clearing house in the end. Necessary facilities should at all times be at his command to pursue the latest diagnostic technic, including the latest bacteriological, microscopical, biological and X-ray tests. Neither from a sense of duty, nor a knowledge of justice, is surgery any longer permissible, without the application of minute diagnostic details.

Diagnosis of abdominal conditions is of the greatest importance and especial stress must necessarily be placed on this subject. The anatomy of the abdomen is important and too much care cannot be given to this region. Topographical anatomy is here of especial importance.

Inspection offers many points of interest in deducing facts. Methods of inspection as to change of position of the patient during the observance should be systematized by each individual physician.

Of the greatest value of all physical signs, palpation stands at the head. Upon a knowledge of proper palpation, in the vast majority of cases, will operation or non-operation depend.

Thoroughness in this part of the work relieves one from many embarrassments later. Nothing should be overlooked. Percussion is used in mapping out various organs of the abdomen, tumors and exudates. It must not be forgotten that distension of the various portions of the intestinal tract, with water or air is often necessary to achieve the best results. Change of position of patient is often of great advantage in arriving at final conclusions,

*Extract of paper read before the Medical Association of the Southwest at Galveston, Texas.

going over the region in question time and again. Especially is inflation useful in diagnosing tumors of the kidney. Vaginal and rectal examination in the female and rectal in the male are always necessary in making a diagnosis of pelvic and abdominal disease.

---

### Observation on Malarial Fever.*

BY L. SEXTON, B. S., M. D., NEW ORLEANS, LA.

The rational treatment of malaria of the tertian type resolves itself into ridding the red blood cells of the asexual plasmodium malariae injected into the blood stream by an infected anopheles mosquito.

No treatment of malaria is worth while that does not take into consideration the eradication of plasmodium in the blood, or protection of the patient against the anopheles mosquito.

Whenever a fever case is visited in a malarial section several blood smears should be taken during the first visit before the administration of quinine, so that with the clinical symptoms a positive diagnosis may be made at once. This is particularly true during times of typhoid and suspected yellow fever, as there is no differential diagnosis harder to make without the microscope than between the malignant forms of malarial, yellow, and typhoid fever.

In tropical countries an examination of the blood for malarial parasites should be made of all subjects suffering from coma, uremia, and dysentery, as the cause of the trouble found early, if malarial, may be the means of saving a life by prompt treatment at the proper time.

The crescent parasites of the pernicious form of malaria are hardly recognizable except by an expert microscopist, after staining, as the evidence is often only small specks of protoplasm with little or no pigment.

The remittent type of malaria is due to the estivo-autumnal form of parasite. The paroxysms are much longer in duration, running into each other so there is no cessation from fever. Such types may be mistaken for typhoid or yellow fever unless the discovery of the plasmodium in the blood or Widal should clear up the diagnosis.

---

*Extract of paper read before Tangipahoa Medical Society of Louisiana.

*Prevention.*—Not within our generation need we expect the eradication of malaria, and this on account of the lack of education and the carelessness of the average physician and parasite carrier. It can be, and has been modified in many communities by the building of residences on the highest part of the farm or town and screening them. Residences should be removed from nearby pools of water and swamps; then by draining and oiling of ponds, or stocking the ponds with numerous fish; by using the essential oils and mosquito lotion on all exposed portions of the body, particularly after night, when the anopheles are most active, many outbreaks of malaria might be prevented. Exposure at night should be avoided, sleeping under a close mesh bar, taking three to five grains of quinine three times a day on alternate days, during the summer and fall months in malarial sections will reduce the infection to one-half or less of the present prevalence.

The night moving picture shows, the night court session, or any gatherings of rural people from malarial sections are among the most common sources of malarial infections.

Drainage and sewerage lowering the water level, the abolition of cisterns in New Orleans has reduced the malaria in this city, until years may pass a busy doctor without having a case unless it is brought into the city or hospitals from the surrounding country.

As said before, three to five grains of quinine on alternate days with meals, during the summer and fall months, is usually sufficient to prevent malaria, and in such small doses can hardly do any harm to those even with an aversion to the drug.

Quinine is germicidal to protozoa by first stimulating and then paralyzing them; from five to fifteen grains daily according to the severity of the case is quite sufficient. Ten grains night and morning on the seventh, fourteenth and twenty-first days succeeding the last chill prevents relapses. One grain daily for each year of the child's age is a very proper dosage for children.

Quinine muriate is quickly absorbed, appears in the urine within fifteen minutes after it is taken into an acid stomach. Its greatest concentration in the blood is within three hours after its administration, hence, ten grains should be given from three to four hours before the expected chill in the adult so as to meet and destroy the young parasites when they are least resistent to its action.

Euquinine is the tasteless carbonized acid of quinine and may be given to children in chocolate, sugar, or milk, and is so insoluble in the mouth that it is almost tasteless in the above mixtures. Its action and the doses are the same as those of sulphate and muriate, only more time is required for its action on account of its insolubility.

When in children, or because of gastric irritation in grown-ups, quinine cannot be retained by mouth, a cleansing enema is given, followed by double the mouth dose of quinine in warm solution held in the rectum by compressing the anus for an hour, or until absorption has taken place. Suppositories of quinine made with cocoa butter are also recommended when it is impossible to retain the drug by the stomach. A preliminary cathartic of some agreeable form should always precede the giving of quinine in malarial cases. The reason many cases hang on for a season, become chronic and cause doctors to proclaim that quinine is not a specific, is the constant reinfection of these patients by working in the same swamps, environments, and fields infested with these infected mosquitoes.

Anemia of malarial and tropical countries is almost universal. Pale people is the rule and not the exception; chronic diseases, hookworm, climate, and food play their part, but the destruction of the red cell by the malarial plasmodium is the principal cause of so much anemia in our Southern country. The cells may run down to two million per c.c., leaving the patient weak and dyspeptic and with all the symptoms attendant upon impoverished blood. Such patient should be kept in bed so long as fever continues and cinchonization should be accomplished as soon as possible to destroy the ameboid parasites. Iron and arsenic, bitter tonics with the most nourishing and digestible diet, bathing, elimination by kidney, bowels, and skin are essential. The patient should be kept in bed, fed on the most digestible food and removed from malarial climate as soon as possible. The ability of arsenic (iron, red bone marrow, and other tonics) to help build up these red cells destroyed by the plasmodium, has caused arsenic to be ranked next to quinine in its curative properties for malaria; but it is the tonic and not the anti-malarial property of the arsenic that accomplishes the good result. Any treatment that would build up red cells as promptly would answer just as well.

It is related of the workers in a mine in Mexico, where the laborers come from the malarial coast country, that though they suffer from arsenical poisoning after working in the mine for some time, they continue to have the chills and arsenical poisoning at the same time, unless quinine is given the chills continue.

So in the treatment of acute malaria no time should be wasted giving arsenic when cinchonization is what is imperatively needed. As a matter of course, arsenic has its place in the after-treatment as a nerve and blood tonic to follow up the eradication of the plasmodia by quinine.

When quinine capsules are given by the mouth they should first be punctured with a needle and followed by some acid drink to insure immediate absorption. Hard compressed pills and tablets may pass through the stomach without absorption and thus cause a failure of cure to be credited to quinine.

There is no occasion in any case to give 100 grains of quinine daily, as it is neither absorbed nor required, and may be toxic in over thirty-grain doses during twenty-four hours. Fifteen grains of quinine makes a 1-5000 solution in the blood of the average patient. Twice this amount is quite sufficient to destroy all the plasmodia (not crescents) if given three days in succession. The quinine acts better when given in two or three, seven to ten-grain doses, preceding the expected chill time by three or four hours, so as to have the maximum saturation of the blood to meet the newly developed plasmodia as they occur in the blood at their most susceptible age to the action of the quinine.

*Contraindications to Quinine.*—Persons with defective hearing, middle-ear disease, and those who are very susceptible and nervous should be given the smallest amount of quinine capable of destroying the germ. Many people have an aversion to quinine and its effect; in others it produces urticaria or disagreeable tinnitus aurium, while others seem much depressed by its use. Such patients should take the quinine in smaller doses or in combination with bromide of sodium or phenacetine, or some other control; but of the evils, quinine effects or malaria, the latter is much more potent for evil to any patient suffering with the disease.

No one should be graduated or licensed to practice who is not thoroughly familiar with the means of cure and eradication of malaria. Boards of health not working on the malarial problem should be superseded by men who know and will combat our greatest enemy. State and national legislatures should be importuned to support liberally all means for the eradication and cure of malaria. Where hookworm or yellow fever has infected one, malaria has infected its thousand.

Our philanthropists can do no better than to help in the eradication of the anopheles mosquito and the drainage and redemption of the lowlands that harbor this parasite. Every drainage canal and reclamation conference is a tack in the coffin of the great malarial scourge of many portions of the United States, for it must be remembered that nearly all inhabitants of our country are liable to the infection of the anopheles mosquito. So it is not local but national in its sphere, and is an important enough subject to command the attention of our greatest national leaders in every State of the Union.

From an economical standpoint the eradication and control of malaria is the most important subject that physicians, boards of health, and drainage engineers have to deal with, for no malarial country can be considered healthy.

---

### A Bold Attempt to Limit the Spread of Venereal Diseases.

In December, 1914, provision was made in the Sanitary Code of New York City for the notification of cases of venereal diseases. The ordinance dealing with this subject is known as Section 88, and reads as follows:

"Section 88. Duty of superintendents of hospitals and dispensaries, and of physicians, to report cases of venereal disease. It shall be the duty of the manager, superintendent, or person in charge of any correctional institution, and of every public or private hospital, dispensary, clinic, asylum or charitable institution in the city of New York to promptly report to the Department of Health the name or initials, together with the sex, age, marital state, and address, of every occupant or inmate thereof or persons treated therein, affected with syphilis or gonorrhea; and it shall be the duty of every physician in the said city to promptly make a similar report to the Department of Health relative to any person found by such physician to be affected with syphilis or gonorrhea. All reports made in accordance with the provisions of this section, and all records of clinical or laboratory examinations indicating the presence of syphilis or gonorrhea, shall be regarded as confidential and shall not be open to inspection by the public or by any other person other than the official custodian of such reports or records in the Department of Health, the Commissioner of Health, and such other persons as may be authorized by law to inspect such reports or records, nor shall the custodian of any such report or record, the said Commissioner of Health, or any such other person divulge any part of any such report or record so as to disclose the identity of the person to whom it relates."

Section 88 has been quoted far and wide as evidence of the aggressiveness of this department in dealing with the problem of venereal disease; but Vermont now comes forward with legislation which strikes an entirely new note and which make our own activities in this field pale into insignificance. The Vermont law, enacted on March 25, 1915, contains the following provisions:

"A person who, knowing himself to be infected with gonorrhea or syphilis marries, shall be fined not more than $500 or imprisoned in the house of correction for not more than two years.

"A person who, while infected with gonorrhea or syphilis, has sexual intercourse shall be fined not more than $500 or imprisoned in the house of correction for not more than one year.

"A physician who knows or has reason to believe that a person whom he treats or prescribes for is infected with either gonorrhea or syphilis, shall immediately report the name, address, age and sex of such person to the Secretary of the State Board of Health, for which report he shall receive the sum of 25 cents, to be paid by the State Board of Health. A physician who fails to make such report shall be fined not more than $200.

"The State Board of Health shall make and enforce such rules and regulations for the quarantining and treatment of cases of gonorrhea and syphilis reported to it as may be deemed necessary for the protection of the public. Said board shall not disclose the names or addresses of persons reported or treated to any person other than a prosecuting officer or in court on prosecution under this act.

"The sum of $1000 is annually appropriated for carrying out the provisions of this act."

This question occurs to us: Will $1000 "annually appropriated for carrying out the provisions of this act" suffice to cover such "quarantining and treatment of cases of gonorrhea and syphilis" as may be "necessary for the protection of the public?" If not, is this merely another case of a paper blockade?—*Weekly Bulletin,* Department of Health, New York.

